# Involving Research Stakeholders in Developing Policy on Sharing Public Health Research Data in Kenya: Views on Fair Process for Informed Consent, Access Oversight, and Community Engagement

**DOI:** 10.1177/1556264615592385

**Published:** 2015-07

**Authors:** Irene Jao, Francis Kombe, Salim Mwalukore, Susan Bull, Michael Parker, Dorcas Kamuya, Sassy Molyneux, Vicki Marsh

**Affiliations:** 1 KEMRI Wellcome Trust Research Programme, Kilifi, Kenya; 2 Oxford University, UK

**Keywords:** Africa, Kenya, data sharing, community consultation, informed consent, governance, trust, ethics

## Abstract

Increased global sharing of public health research data has potential to advance scientific progress but may present challenges to the interests of research stakeholders, particularly in low-to-middle income countries. Policies for data sharing should be responsive to public views, but there is little evidence of the systematic study of these from low-income countries. This qualitative study explored views on fair data-sharing processes among 60 stakeholders in Kenya with varying research experience, using a deliberative approach. Stakeholders’ attitudes were informed by perceptions of benefit and concerns for research data sharing, including risks of stigmatization, loss of privacy, and undermining scientific careers and validity, reported in detail elsewhere. In this article, we discuss institutional trust-building processes seen as central to perceptions of fairness in sharing research data in this setting, including forms of community involvement, individual prior awareness and agreement to data sharing, independence and accountability of governance mechanisms, and operating under a national framework.

Increasing scientific access to data generated through research on public health is widely seen as an important, or even essential, way of strengthening the utility of research and promoting public health interests (Manju & Buckley, 2012; Pisani & AbouZahr, 2010; Toronto International Data Release Workshop et al., 2009). Important ways in which this could happen include promoting reproducibility, efficiency, and generalizability of public health research. Many science funders and standard setters now require the inclusion of data-sharing plans in funding applications (Economic and Social Research Council [ESRC], 2010; Medical Research Council [MRC], 2014; National Institutes of Health, 2014; Wellcome Trust, 2010), and high profile scientific journals sometimes require the publication or at least archiving of the data sets that inform published papers (Nature, 2014; PLoS Journals, 2014; Science, 2015). This move has been seen as particularly important for certain types of high utility research data sets (Biotechnology and Biological Sciences Research Council [BBSRC], 2010; Manju & Buckley, 2012) but applicable to all.

## Practical and Ethical Challenges to Data Sharing

Researchers and research institutions have been relatively slow to take up data-sharing policies in practice (Manju & Buckley, 2012; Nelson, 2009; Piwowar, 2011). Issues have been described for different research stakeholders, primarily for originating researchers (those who undertake primary research generating data) and study participants and the communities to which they belong, but also for funders, publishers, clinicians, publics and private sector interests (Foster & Sharp, 2007). An overarching issue for data sharing is that the potential benefits and challenges, practical and ethical, are not easily generalized but depend on specific details of the type of data, who is requesting use and for what purpose (Foster & Sharp, 2007; Pearce & Smith, 2011; Pisani, Whitworth, Zaba, & Abou-Zahr, 2010; Sankoh & Ijsselmuiden, 2011).

The lack of specific guidance for research data sharing may be an important limit on researchers and research institutions’ willingness and ability to share data more widely (Manju & Buckley, 2012). Dominant challenges in data sharing include the following: how to respect study participants’ autonomy when future uses of data are unknown at the time of an original study; ensuring privacy, including challenges for de-identification of study participants and communities; risks of intentional or non-intentional misrepresentation of data by requestors, with risks to scientific validity and potential for harm to participants and their communities; risks of undermining originating researchers’ professional development; and challenges in accessing the resources needed to support data sharing (Bull, Roberts, & Parker, 2015; Coady & Wagner, 2013; Manju & Buckley, 2012; Mello et al., 2013; Pearce & Smith, 2011; Pisani et al., 2010; Sankoh & Ijsselmuiden, 2011).

### Context

The context in which the original research was conducted is also seen as a potential influence on ethical policy, with particular issues for global sharing of data collected during studies in low-to-middle income countries (Pisani et al., 2010; Sankoh & Ijsselmuiden, 2011; Tangcharoensathien, Boonperm, & Jongudomsuk, 2010). Data sharing in this context risks widening underlying global inequities in access to resources for research and health, raising the need for special protections, such as time-limited embargos, to give protected access to originating researchers (Parker et al., 2009; Rani, Bekedam, & Buckley, 2011). For the same reason, initiatives to build analytic capacity in originating research teams, including through collaborations with requestors, have been seen as centrally important. Across all of these challenges, although risks are not easily quantifiable, an important underlying issue is one of maintaining the trust of researchers, community stakeholders, and the wider public. Failure to do this may generate negative implications for willingness to share data in the short term and for longer term relationships needed to support research, as has been described particularly but not only for bio-repositories (Foster & Sharp, 2007; Hawkins & O’Doherty, 2010; Pearce & Smith, 2011).

### Fair Policies and Practices

These challenges highlight the need for fair processes in data sharing, that is, processes that are locally responsive and justifiable within widely recognized ethical principles (Fullerton, Anderson, Guzauskas, Freeman, & Fryer-Edwards, 2010; Hawkins & O’Doherty, 2010; Pearce & Smith, 2011). Of particular importance, there are strong arguments for more data sharing in low-to-middle income countries especially for efficient use of limited resources, but in many such settings, policies are lacking (Manju & Buckley, 2012). To date, there is relatively little empirical evidence on what constitutes fair process in research data sharing from the perspective of research stakeholders, particularly from low-to-middle income countries. In response to these challenges, this qualitative study was designed as part of a wider project exploring the experiences and views of a range of stakeholders in international research in Kenya, India, Thailand, Vietnam, and South Africa, funded by the Public Health Research Data Forum (http://www.wellcome.ac.uk/PHRDS; Bull, Cheah et al., 2015; Cheah et al., 2015; Denny, Silaigwana, Wassenaar, Bull, & Parker, 2015; Hate et al., 2015; Merson et al., 2015; Parker & Bull, 2015).

The present article describes research designed as a consultation on data sharing, mapping the views and values of diverse stakeholders in a large international research program, the Kenya Medical Research Institute (KEMRI) Wellcome Trust Research Programme (KWTP; http://www.kemri-wellcome.org). This article focuses on views on “fair processes” in data sharing. Findings on the benefits, challenges, and acceptability of data sharing will be published separately.

## The Research Program

KWTP is a large international research program, initiated as a collaboration between KEMRI and the Wellcome Trust in 1989. KEMRI is a national parastatal organization mandated to conduct medical research in Kenya, established through the National Council for Science and Technology (Amendment) Act of 1979. The KWTP research focuses on health issues relevant to populations in Kenya and similar settings, including clinical, epidemiological, basic science, health systems, and social science research. Research is conducted in several sites in Kenya and through a large number of scientific collaborations across sub Saharan Africa and globally. The program headquarters are in close proximity to Kilifi County Hospital, and researchers work in strategic partnership with local Ministry of Health policymakers and providers. Research activities in Kilifi include the running of a Health and Demographic Surveillance Survey in the county hospital’s catchment area to support research and health service delivery, covering a population of 260,000.

The KWTP is a member of the INDEPTH Network (2013) of health and demographic surveillance sites in Africa, and works with the Kilifi Ministry of Health to collect and utilize routine clinical surveillance data in the County Hospital and surrounding health facilities. A large and complex mix of research data are archived within the KWTP and, at times, in external repositories. Given the multi-disciplinary nature of the program, these data have been generated through a range of research projects and linked demographic and clinical surveillance activities over time. Research governance is through an institutional scientific coordinating committee, KEMRI’s National Scientific and Ethics Review Committees, and where required (e.g., by funders) through other international research governance bodies.

To date, there is no national research governance policy guiding public health research data sharing in Kenya, although dialogue is beginning to take place in KEMRI (V. Marsh, personal communication, Feburary, 13 2015). The Kenyan Data Protection Bill (2013), which includes people’s right to know how data collected about them will be used, is in the process of being passed into law but does not address research data sharing (Data Guidance, 2014).

## Method

The study methods, including for data collection and analysis, were developed through a series of international workshops involving partners in the multi-country study (Parker & Bull, 2015). An overall aim was to balance the development of a common approach to strengthen transferability of findings across countries and the need to take account of important contextual features in each site. In Kilifi, methods drew particularly on existing community engagement activities in the KWTP and earlier experience with community consultation on relatively novel and complex issues in research, as described below. Given the nature of major research data sets in the program, the scope of the consultation included sharing data from research and from research-related demographic and clinical surveillance activities.

### Study Area

The study was conducted in the Kilifi Health and Demographic Surveillance System catchment area in Kilifi County on the coast of Kenya. This area has a mixed community, including some of the most poor areas in Kenya, especially in rural locations (Chuma, Gilson, & Molyneux, 2007), and the county government headquarters, a thriving university, and the KWTP in Kilifi town. The majority ethnic group is the Mijikenda (Parkin, 1991); subsistence farming and fishing are main livelihoods, with an emerging urban service industry. Community engagement includes regular consultation with a network of approximately 200 KEMRI community representatives (Kamuya, Marsh, Kombe, Geissler, & Molyneux, 2013; Marsh, Kamuya, Rowa, Gikonyo, & Molyneux, 2008). KEMRI community representatives are typical residents selected by local communities at public meetings to undertake a consultation role for 3 years. Annual participatory trainings, including basic research and research ethics as topics, and regular meetings support community representatives, capacity to input on research-related topics. A full description of the program, community engagement, and the surrounding community is given elsewhere (Marsh et al., 2008).

### Study Participants

The study involved participants with varying degrees of research experience. These included researchers (junior, mid-career, and senior) with high levels of research experience (12 individuals), program health providers and research frontline staff (community facilitators and field-workers) with variable but generally less research experience (18 individuals), and more typical community members, including assistant chiefs and community representatives, with relatively low research experience (30 individuals). Field-workers are program staff who support research activities, including obtaining informed consent and collecting data and biological samples (Kamuya, Marsh et al., 2013). Field-workers and community facilitators originate from the community, have at least 12 years of formal education, and receive training on research methods and research ethics. Study participants were selected purposively to maximize diversity in age, gender, and professional roles. For community representatives, diversity in educational status, residency in urban or rural settings, religion, and gender balance were also considered. Table 1 summarizes participants’ characteristics.

**Table 1.** Characteristics of Participants.

Participants	Total	Age range in years	Men:women	Education range in years	Religion
Research staff	14	30-59	10:4	5 to ≥30 post basic	Christian (8) Muslim (3) Other/none (3)
Staff: Field-workers	11	28-45	6:5	12-16	Christian (11) Muslim (0)
Staff: Community facilitators	5	30-51	3:2	12-18	Christian (4) Muslim (1)
Assistant chiefs	6	33-50	3:3	11-12	Christian (5) Muslim (1)
KEMRI community representatives	24	26-81	15:15	None (1) Informal (1) ≤8 years (primary) - 10 ≥9 years (secondary) - 12	Christian (22) Muslim (2)

*Note.* KEMRI = Kenya Medical Research Institute.

Box A. **Vignette and Question Guide for Community Stakeholders’ Discussions.**

A researcher working with another *government research institution in Kenya* needs data from KWTP to try to find out whether there is any association between having good access to water supplies and chances of being admitted to hospital for diarrhea. Results from this work will help to come up with better ways of preventing diarrheal diseases in the community the researcher is working with. The researcher is working in an area which is very like Kilifi but does not have basic census or hospital surveillance systems. The information he or she is requesting is (a) all clinical information collected about individuals admitted to hospital in the last year (explain what this is) and (b) what kind of water supply they have at home (from census).

Should this information be shared? Why/why not? What, if anything, could be done to make data sharing more acceptable?

What if the researcher is working at another institution/non-governmental organization?

What if they come from outside Kenya/outside Africa?

What if they want the information for research on health conditions not common in Kilifi/Kenya?

### Data Collection

Data were collected between January and June 2014. The methods used for more and less research-experienced participants varied, reflecting the extent of information sharing about research and data sharing needed to support informed debate.

#### For researchers and health providers

We held 1- to 2-hr in-depth interviews, drawing on individuals’ data-sharing experiences. Given the varied background of individuals, discussions on data sharing related to differing types of primary research, including clinical trials, basic science, qualitative research, and clinical and demographic surveillance.

#### For community facilitators, community representatives, assistant chiefs, and field-workers

We held extended small group discussions (5-6 people), using a case study to inform and focus reflection. The case study focused on an integrated database of Health and Demographic Surveillance and hospital-based clinical surveillance data routinely used by the Ministry of Health and researchers in Kilifi to support health service delivery and research (Cowgill et al., 2006). In these groups, facilitators introduced a series of questions, framed as a vignette (see Box A), about who else might reasonably request access to data, while varying geographic location and proposed uses of data. For assistant chiefs and community representatives, visual aids were used to support presentation and discussion of the case study. The nature of this case study meant that a wide range of individual and household level data were considered in these discussions, including household geo-positioning and individual demographic, clinical, and laboratory data.

Group discussions were held in Kiswahili, Kigiriama (local language), or English depending on participants’ choices and lasted 3 to 4 hr inclusive of a short break. The methods were developed through earlier program community consultation activities to facilitate debate around unfamiliar complex topics (Marsh et al., 2013; Njue, Kombe, Mwalukore, & Marsh, 2015). The approach draws on principles in deliberative forms of ethics (Parker, 2002) and related forms of deliberative consultation (Hawkins & O’Doherty, 2010). After each group discussion, three or four individuals were selected for follow-up interviews at home, lasting 30 to 45 min, to assess the stability of views over time. Individuals were purposively selected for follow-up interviews to reflect differences in gender and attitude, including support for or rejection of data sharing, and to further explore views of those making minimal contributions to discussions.

*Across all interviews and group discussions*, facilitators built on participants’ knowledge and experience to provide inputs and explore views on potential benefits, challenges, and good practice, and used careful probes on ethically relevant issues. Tools used for in-depth interviews and group discussions are included as supplementary files (available at http://jre.sagepub.com/supplemental).

### Data Management and Analysis

All interviews and discussions were audio recorded, transcribed, and translated where needed. For group discussions, detailed notes were taken and debriefing sessions held by facilitators to discuss emerging findings.

Data were analyzed using a framework analysis approach (Green & Thorogood, 2007), working with themes drawn from topic guides and emerging from the data. In this analysis, broad themes were related to factors influencing acceptability of data sharing and views on consent and governance processes. I.J. and V.M. closely read all transcripts for data familiarization, developed an initial coding framework, and coded transcripts iteratively using Nvivo 10 software (QSR International). Analysis charts were developed to collate individual- and group-level views across themes. Separate charts were used to describe the flow and dynamics of discussions (e.g., change of views over time).

Throughout data collection and analysis, facilitators were aware of and consciously aimed to minimize potential influences on views expressed, including from their own positionality and from interactions within groups, challenges typical of qualitative research (Green & Thorogood, 2007). Facilitators’ positionality was considered in developing tools and in collecting, analyzing, and interpreting data. For example, a key issue was that perceptions of the alignment of facilitators with the research program might limit open criticism of policies, including for data sharing. Strategies used included holding group discussions within the community, away from the research center; maintaining a neutral position on topics under discussion; ensuring positive and negative implications were discussed; and emphasizing the role of the consultation in informing future policy.

I.J., F.K., and S.M. were mainly responsible for facilitation of group discussions and V.M. for individual interviews. I.J., F.K., and S.M. are experienced facilitators who originate from this community; V.M., D.K., and S.M. have lived in Kilifi for more than 18 years. Analysis was conducted mainly by I.J. and V.M., supported by the other authors through an iterative process, including cross-checking data coding and input to analysis charts.

### Ethics Review

The study was approved by KEMRI Scientific Steering and Ethical Review Committees in Kenya, and under a parent protocol covering all country sites by OXTREC in the United Kingdom (OXTREC 1051-13). All participants gave individual written informed consent for participation, for audio recording of interviews, and for data collected during this study to be shared with other researchers in an anonymized form.

## Findings

Given the aim of this article to inform fair processes in data sharing, the main findings describe stakeholders’ views on informed consent, decision making about access to data, and forms of community engagement. Two overarching and interrelated issues importantly inform these views. First, views were strongly influenced by familiarity with data sharing and the associated debates in the literature, which as expected was greater among more research-experienced stakeholders. Less research-experienced stakeholders generally had little awareness that researchers might share data within the scientific community, how this might occur, and what uses this might have for progress in science. This meant that discussions with community stakeholders involved greater input from facilitators on the reasons for sharing data and probing around potential challenges.

Second, across all discussions, perceptions of fairness in data sharing were importantly informed by reflection on the potential benefits and challenges that might be entailed. These issues will be discussed in more detail in a separate publication but are summarized below.

### Overview of Perceived Benefits and Challenges for Research Data Sharing

A majority of stakeholders were generally supportive of data sharing in principle, but important conditions were proposed by all, discussed in the following sections. Overall, most concerns and cautions were raised by community representatives, although to some extent these views shifted toward being less prescriptive with increasing understanding of the potential value and protections that could be implemented for data sharing. Across all stakeholders, many challenges reflected those in the literature, including for study participants, communities, and originating researchers. Particular challenges for study participants and communities included risks of loss of privacy and stigmatization, where stigmatization was seen as more closely linked to the use of data than to its nature. For example, socioeconomic data associated with poverty, such as poor access to sanitation, were seen as potentially stigmatizing across communities. Importantly, many were concerned to see potential for health benefits for primary communities from new research, recognizing low access to health resources across this community. For originating researchers, challenges included a potential to undermine professional development where better-resourced researchers gained access to data before planned analyses were completed, risks to scientific validity through intentional or unintentional misuse of data, and challenges in obtaining resources to support effective data sharing.

In this way, there were concerns that the interests of stakeholder groups in this setting should not only be protected but also be promoted as far as possible. As described in the literature, this focus was directly linked to recognition of underlying global structural inequities in access to scientific resources (for researchers) and to health resources (for primary communities). A strong emerging theme was the need to ensure that data-sharing practices did not contribute to widening these differences but, as far as possible, promoted equity (Manju & Buckley, 2012; Pisani et al., 2010; Sankoh & Ijsselmuiden, 2011; Tangcharoensathien et al., 2010). On this basis, data sharing was most strongly supported for new research where benefits could be derived from a direct influence on policy and practice in this and similar low-to-middle income settings.

### Informed Consent Processes

#### The importance of individual consent

Across all stakeholder groups, there was strong concern that research participants should understand that study data related to them might be shared in future and were given an opportunity to accept or refuse this component of research. Acceptable *types* of consent are discussed later, but some form of prior individual understanding of, and agreement to, data sharing was seen as important by a majority of stakeholders, with variation across groups. Among community stakeholders, this prior understanding was, with few exceptions, seen as essential in data sharing. Among researchers and health providers, prior agreement to data sharing was seen as important, but for many, this was part of a set of issues to be balanced in given situations, including scientific gains and the existence of any potential for harm or loss of privacy to participants or primary communities:

I can only say it is good for them to know, but personally I wouldn’t be very worried if what they didn’t know is not harming them. (IDI05, mid-career researcher, male)

Given the novelty of data sharing, all stakeholders recognized important communication challenges in trying to convey complex new information as part of a consent process for an ongoing study. This challenge was interpreted by a few, including community stakeholders, as a reason to be cautious about giving any information at all on data sharing. But a majority saw, instead, a need to invest resources in explaining data sharing carefully as part of study consent processes. Two main reasons underpinned this view. First, it was seen as wrong to use data in ways that people would not have agreed to if they had known, on the basis of failing to show respect and denying participants a reasonable right to “say no.” Second, if covert data-sharing practices later became known, this could present major risks to trust, the sustainability of relationships between researchers and the community, and, therefore, the research itself.

I must know . . . that my . . . information will be shared for it to be used in research. But there are others who won’t agree. So, according to me it’s good to be informed so that if you don’t want you will indicate it right there in the form. (R1FGD5, community representative, male)

. . . you might assume that this one is just a community member . . . he will have no way of seeing this data again . . . but he might come across that information, then he’ll come to us . . . saying “Okay . . . is this what you are doing?” Now he’ll tell you this is the end of me giving any information when you come to my place . . . and you know he can spread that. (R3FGD3, community facilitator, male)

The likelihood of variation in attitudes to data sharing underlined the importance of a right to refuse. Individuals were seen as likely to have different views on the acceptability of data sharing in general and in relation to specific sensitivities linked to types of data. Illustrating variation in the perceived sensitivity of genomic data, a mid-career researcher explained,

[I think it would be fine] if someone maps everything about me [laughing] and they could probably figure that in 10 years I will die of a certain disease that I don’t know I have! But someone else might not be comfortable with that. (IDI02, mid-career researcher, female)

Given difficulties in explaining data sharing, many researchers and frontline staff were concerned that consent could become a “tick box” exercise. In practice, the main concern for researchers and health providers was to provide potential participants with an opportunity to opt out of data sharing, seen by many in this group as more appropriate than an “opt in” approach. For some stakeholders across all groups, the consent process was also seen as having a wider educational role to explain the potential scientific value of data sharing in general. In addition, building understanding of processes for data sharing might generate more confidence in research and data sharing, including through this active demonstration of openness.

#### Types of consent

Across all stakeholders, discussions on the most appropriate forms of consent for future data sharing commonly reflected a need for compromise between wanting consent to be more specific (to strengthen choice) and the limitations this could place on potential utility of data. In an ideal world, many stakeholders felt that consent processes should involve individuals understanding how, why, and by whom their data would be used in future. With recognition that this information would not be known at the time data were collected, options prompted by facilitators for discussion were placing limits around types of future use during prior consent, re-contacting study participants to seek consent for a new use, and using a broad form of prior consent to unspecified future use (Sheehan, 2011). All of these options could be tied to governance mechanisms, discussed later. There was eventual general consensus across groups that *broad forms of consent would be most appropriate*, without specific limitations on future use but with additional forms of governance over access decisions, as discussed in the following section. This position was generally reached after considering the “pros and cons” of different consent options.

*Re-consenting processes*, involving re-visiting research participants to seek consent to specific forms of data use in future, and following broad consent processes, were widely seen as impractical, particularly for large studies, over longer periods of time and where data were fully anonymized. A mid-career researcher noted that re-consenting might be a very insensitive practice, for example, if the individual involved had subsequently died. Among community representatives, several commented on the likelihood that re-consenting had particular risks of generating concerns and questions, especially over time where participants might have forgotten giving broad consent for data sharing:

[The person] just knows these are KEMRI people and they have come to ask me questions and after that they don’t care that KEMRI staff have explained to us this and that . . . when you go back later on and ask them . . . they will tell you that they don’t remember you and so you will have to start afresh up to the end. (R6FGD8, community representative, female)

For these reasons, some community representatives described re-consenting as an unnecessary inconvenience to people.

As another option to broad consent, there was little support for the idea of *placing restrictions around future use of data* at the time of its collection. For all stakeholders, there were challenges in imagining what these restrictions might usefully be. Some researchers also felt that “conditions” might be unreasonably used by originating researchers to control data-sharing potential in their own interests.

As before, some researchers saw specific prior informed consent as less important, underpinned by an attitude that its fundamental value meant that data sharing should be presented as a normal and important element of research. In this view, the onus was on researchers to make sure participants understood the possibility of unspecified future data sharing, and to offer clear and positive explanations about policy as part of the background to the research program and its ways of working. Further illustrating this viewpoint, a senior researcher saw broad consent with minimal governance as a most honest approach given practical challenges in controlling requestors’ use of data after sharing.

### Oversight for Decisions on Access to Data

Across all researchers and health providers and many community stakeholders, there was wide and strong agreement that effective processes would be needed to provide oversight for decisions about future requests for research data sharing, particularly where broad forms of consent had been used. While researchers, health providers, and a few community stakeholders saw some forms of data as requiring less oversight, particularly aggregated, anonymized, and “less sensitive” data (such as routine demographic data), a reasonable oversight process was still needed to determine which data could be made more openly accessible. In any case, sensitivity of data was more often seen as related to its use than its nature. More research-experienced stakeholders, including frontline staff, described provision of oversight as an ethical responsibility for originating researchers, in relation to their study participants and communities:

I think we have responsibilities to all those groupings [stakeholders]. I would prioritize in some senses the individuals who [participate], I think it’s such a fundamental concept behind the idea of ethical research, that “do no harm,” and harm could be widely interpreted. And most of the things we’re concerned about, things like loss of anonymity or stigmatization, are harms. So that should be a starting responsibility, I think. (IDI10, senior researcher, male)

Among community representatives, these views emerged over the course of discussions with increasing understanding of the complex ways in which data might be used in future, the implications of this, and the nature of broad consent. Many researchers more clearly articulated the problem as being able to respond fairly to requests when these could not be predicted in advance, and where a reasonable decision was likely to depend on specific features of context, including what data were being requested and how they would be used. Without context, as a senior researcher typically described, “These questions are almost unanswerable, because there are times when maybe the greater good overwhelms the individual priority” (IDI04, male).

In addition to making decisions about whether requests for access were reasonable, more research-experienced stakeholders reflected on a potential need to recognize and account for the resources invested in collecting, storing, and sharing data through processes of prioritization. Many researchers and some community stakeholders queried the extent of their responsibility to support data sharing, given these resource costs, and most felt that these activities could adversely affect the potential of researchers in the program to conduct new and important research:

What obligation is there to spend a lot of people’s time and effort cleaning, obtaining and making sure data are accurate for a question which you did not set out to answer, and does not seem a very good or relevant question? (IDI01, senior researcher, male)

While many considered that oversight mechanisms were essential for data requests, limitations were also recognized. All stakeholders felt these processes would still work largely on the basis of trust that requestors would comply with terms of data-sharing agreements and not use data in other ways including passing them on to third parties. For this reason, researchers and health providers made strong recommendations that data sharing should as far as possible take place within collaborative partnerships to strengthen protections for primary communities and originating researchers. An additional benefit of scientific collaborations seen by some community stakeholders was a potential to bring new research to the community, with concomitant direct and translational benefits. But illustrative of concerns about the nature of trust, a community representative who opposed data sharing throughout this consultation did so on the basis of challenges in maintaining control in the long term:

. . . for instance if anyone gives me his shirt now, after that even tomorrow if he sees the shirt is dirty because I don’t wash it, he cannot say anything . . . now it belongs to me, I can also give it to someone else . . . So I am saying that when we agree . . . that means you are in agreement with whatever the other person is going to do. (R1FGD8, community representative, male)

#### The make-up of an oversight group

For all stakeholders, the acceptability of broad consent processes was tied to the functioning of a group trusted to make decisions about future data requests by fairly balancing the interests of different stakeholders. Many saw risks in individual researchers making these decisions alone:

I would suggest the researcher should not be the only person . . . able to release this information. There have to be at least good policies . . . and the funders need to have approved . . . and either to have a body . . . like a committee . . . that will help it be approved and check on . . . technical matters. (R2FGD2, field-worker, male)

There was considerable debate about the make-up of an oversight group and how it should work. The least research-experienced stakeholders were least certain about these processes, quite commonly expressing surprise at being asked and feeling unable to comment on appropriate processes. This group often referred to following strict “rules,” for which the KWTP was often seen as a benchmark, without explaining these rules or how they should be applied to data sharing:

. . . but maybe what you can do is that if there are any rules that are to be followed by the person requesting information then he should abide by them. (R4FGD5, community representative, female)

Across all groups, the most common suggestions for governance defaulted to knowledge about the existing institutional- and national-level scientific and ethics processes. More research-experienced stakeholders suggested that oversight groups should include scientific, ethical, and legal expertise, and lay and professional perspectives. Originating researchers would be needed given their understanding of the data and how they were collected. Particularly for community stakeholders, Ministry of Health representatives were seen as important in providing necessary checks and balances, especially for international forms of data sharing:

I think there should be the committee (to oversee decisions) but the government must also know . . . It should be informed . . . then both the national and the County governments will be aware . . . that there is so and so who wants to do this and that. (R3FGD6, community representative, male)

An important and controversial area in this debate was whether and how community stakeholders should be represented in the governance of data sharing. Stakeholders across all groups felt community representatives should be involved in developing a mutually acceptable data-sharing policy, described as a “shared vision” for research (IDI07, senior researcher, male). Furthermore, it was seen as important that people within the community *did not feel excluded* from these processes. Many also saw it as ideal to include community representatives within an institutional oversight committee:

I think . . . this body should not just be left to people with expertise alone. I think we should also have . . . representation from the community where this data is generated, so that they can be part of these decisions and see how the data is used . . . I think it would bring more trust from the community that “I was there” . . . . (IDI12, nurse manager, female)

At the same time, for all groups, there were two main challenges to this approach. First, it might be difficult to ensure meaningful input to debates given the complexity of the topic, including the nature of widely different forms of data and their uses. Second, there was a challenge in identifying which individuals should participate and in what way they would be representative of the wider community or the individuals who had contributed data. There was a concern that inclusion of one or a few community members in a part of this process could become tokenistic. Others felt uncomfortable that a few community “representatives” might overturn the decisions about data sharing that individual participants had made:

Let the . . . the leaders . . . be given information . . . I didn’t say that they [leaders] should be asked whether they agree or refuse because . . . participants . . . already gave consent for the information to be shared . . . . (R4FGD4, assistant chief, male)

Across all groups, stakeholders emphasized the importance of independent oversight of data-sharing processes within the program, currently met through linkages to national-level ethics review processes. On this basis, while many community facilitators and field-workers felt they could represent community perspectives adequately, this view was countered by concerns about their independence.

On direct probing, several community stakeholders saw some potential in working with additional community-based groupings to supplement an institutional committee, including chiefs, religious or other leaders, and community representatives as members. These views were not strongly voiced, often seemed exploratory, and sometimes generated cynicism about the capacity of community stakeholders to “refuse” decisions already supported at a high level in government. All groups saw a need for more research and debate to formulate proposals for greater community involvement.

#### Balancing accountability and efficiency

A key consideration for most researchers and health providers, many of whom had experience of requesting and sharing data, was that governance mechanisms should balance transparency and accountability with efficiency of process. Researchers were keen that data-sharing processes should not be turned into a “bureaucratic nightmare.” Many referred to experiences in the past when accessing data from other researchers had been an easier process, often based on personal requests to known collaborators or colleagues. These views were implicitly critical of these less formal decision-making processes as lacking standardization and accountability, and being sometimes entirely subject to the wishes of originating researchers or relationships between requestors and originating researchers. There was at the same time some “guilty” nostalgia for the ease of these processes in many instances:

We’re coming from a situation where people held data on their computers as personal pieces of projects, it’s really hard . . . Before we’d just go to someone from the [research] group . . . they would happily give it out . . . But now we go, make the request, come back . . . then they send you to that same data manager you would probably have just gone straight to . . . . (IDI02, mid-career researcher, female)

### Community Engagement

Among all stakeholders, a majority strongly felt that community engagement would be an important element of ethical practice for research data sharing. In addition to involving community representatives in decision-making processes for data access requests, described earlier, other forms of community engagement included (a) creating wide awareness of data-sharing practices within the KWTP, including benefits and the ways in which any risks (e.g., identification of individuals) would be managed; (b) giving feedback to community members or representatives on data-sharing activities over time; and (c) involving community members in consultation activities about specific data-sharing requests on an ad hoc basis.

#### Creating general awareness of data-sharing activities

For all stakeholder groups, generating a balanced view about data sharing that included awareness of choice across the wider community was seen as important to the project overall. This awareness of data sharing was described as potentially more important than explanations during individual consent, given differences that might arise at study level and greater risks of tokenism for the latter. Individuals’ own levels of positivity about data sharing were often directly reflected in the way they anticipated community engagement working:

I think what you need is a straightforward statement about the role of KEMRI, the kind of data that KEMRI collect, and how we see the best way that those data are used. (IDI07, senior researcher, male)

Another researcher, a strong active advocate for open access, proposed that community engagement processes should be “just shy of evangelical” (IDI06, senior researcher, male).

At the same time, all stakeholders clearly recognized the challenges of engaging wider communities on data sharing in a way that would create adequate understanding of a complex technical subject with inherent uncertainties, highlighting a need for specific investment in communication and operational research. These challenges for engagement, and examples of approaches to tackling these, are reflected in this study. Engaging communities widely would inevitably carry risks of rejection or generating negative attitudes, with potential to undermine the acceptability of research across the program. For this reason, a minority of stakeholders, including community representatives, felt that the risks of misunderstanding were too high and important for wide community awareness–creating activities to be undertaken.

But if you suggest going to tell them that people are requesting this [data] . . . the negative concerns that people had will then come up again and . . . we don’t know where we will be heading to. (R2FGD5, community representative, male)

Some frontline staff suggested that a gradual process of introducing these concepts, beginning with individual informed consent, would help to reduce the risks of widespread misunderstanding.

#### Giving feedback on data-sharing process

To counter concerns about loss of autonomy and trust in broad consent processes, several more and less research-experienced stakeholders commented on the importance of regular feedback to communities on data-sharing activities over time. This was seen as a way in which researchers could ensure that community voices provided local checks and balances to policy, maintain some level of transparency and community accountability, and avoid later loss of trust where particular instances of data sharing produced controversy:

Let the national committee and the committee from this center consent, but let the leaders of the people who were involved in that research at that time be . . . informed, for them to expect findings from that country. (R4FGD4, assistant chief, male)

At the same time, giving feedback on data sharing could be undertaken alongside feedback of research findings more generally, and would avoid the challenges of understanding how to include community representatives in data access decisions. Feedback processes were, however, recognized as having the same challenges of representation and communication as other forms of community involvement.

#### Community consultation

Consulting community stakeholders on data-sharing policy, as represented by this study, was seen as an important form of community involvement. As before, many of the more research-experienced group pointed out that community perspectives should place absolute limits on practices of data sharing:

. . . through discussions, a shared vision of that [data sharing policy] can be created with the community. (IDI 07, senior researcher, male)

Similarly, some respondents from all groups raised the possibility that community groups could be consulted about particularly challenging requests, although which areas might present such challenges were not clearly explored in these discussions.

## Discussion

The study has engaged a range of research stakeholders in the KEMRI Wellcome Trust Research Programme, through in-depth discussions on fair processes for sharing research and routine clinical and demographic surveillance data within the scientific community. Emerging views on good practice were importantly linked to attitudes to data sharing overall, particularly perceptions of risk and benefit. The uncertainty inherently involved in data-sharing practices, about who might ask for data, and for what purpose and when, generated a set of conditions around data sharing. While more and less research-experienced stakeholders largely expressed support for the idea of sharing data, these conditions were foundational to acceptability. The main principles underlying these conditions relate to *autonomy rights*, the need to ensure that *stakeholders’ interests are not only protected but also promoted* as far as possible, and the related importance of *trust in researcher–community relationships*. These principles, and the recommendations on practice that they generated, are discussed in the following sections, linked to the literature on institutional trust-building mechanisms.

Overall, we note that the careful deliberative processes used to empower different stakeholders to contribute to this consultation illustrate that it is feasible, although not straightforward, to seek substantial community or public input on data-sharing policies broadly, as has been shown in high-income settings (Hawkins & O’Doherty, 2010). Further, from our observations of process, that the extent of understanding of the aims and processes for research data sharing strongly influences attitudes (Marsh, Kamuya, Parker, & Molyneux, 2011) including potentially generating unrealistic expectations of benefit and risk. However, it has been important for us to take account of limitations that may be related to the methodology used. Deliberative forms of public consultation can generate particular challenges in influencing views expressed, particularly through “biases” implicit in the attitudes of facilitators and within-group dynamics (Burgess, 2004; Hawkins & O’Doherty, 2010). At the same time, information sharing and the development of informed viewpoints have formed an essential basis for this consultation. Throughout the planning and conduct of the study, we have maintained high awareness of and sought to limit these influences, as described in the “Method” section of the article.

### The Rights of Individuals (and Communities) to Know About Data Sharing

Autonomy rights were related to individuals’ rights to know about the practice of data sharing in a general sense, that is, its value, potential benefits and risks, and mechanisms of governance. Two types of activities were recognized as important to support these rights: individual informed consent processes and community-wide engagement.

In relation to informed consent, assuming that the privacy of study participants and primary communities would be protected, there was general agreement that good practice would include a *broad understanding and agreement to future data sharing*. However, it was agreed that this “broad understanding” should be presented simply given the challenges in seeking more traditionally specific types of informed consent at the time of data collection. Of note, in this setting, an alternative strategy of re-consenting was seen as likely to generate confusion, anxiety, and inconvenience for study participants and communities. Importantly, the acceptability of broad consent was based on compromise and linked to a further condition that future requests for data access would be carefully reviewed through a fair oversight process. This view is supported by arguments in the literature on genomics data that broad consent can sufficiently support autonomy in informed consent by acting as a decision to allow others to decide. In this case, different types of information may be needed. It has been argued that broad consent processes require that less information is given on future uses of data, and more on the benefits and challenges of sharing the particular data involved, and on how challenges will be addressed, including processes by which decisions are taken (Sheehan, 2011).

Community-wide awareness of data sharing was seen as critical to support individual consent processes but challenging to provide. Difficulties in ensuring sufficient understanding of primary research through informed consent are well recognized, including in this setting (Participants at an International Workshop on Informed Consent and Community Engagement, 2013). Giving additional information on data-sharing practices at this time would be challenging in its own right (as demonstrated throughout this consultation) and would additionally risk undermining understanding of the primary research. Community engagement is already seen as a mechanism to support individual informed consent processes at the KWTP, and engagement over data-sharing practices would be in keeping with this approach. Creating community-wide awareness of data sharing would require the investment of significant resources in careful piloting, rolling out, and evaluating of process. Widespread misunderstanding and loss of trust in researcher–community relationships could otherwise be a serious consequence. Importantly, where data sharing becomes a common practice, loss of trust may also result from a failure to adequately inform individuals and communities about this activity. Furthermore, community engagement strategies that build on community-wide awareness of data sharing are important in relation to other principles, as discussed in the following sections.

### Protection and Promotion of Stakeholders’ Interests in Low- to Middle-Income Countries

A strongly emerging view in this consultation was that the interests of primary communities and originating researchers should not be harmed through data sharing, but should as far as possible be promoted. Promotion of interests was framed as an ethically important means of reducing, or at least not increasing, structural forms of existing global inequities between better and less well-resourced areas of the world (Manju & Buckley, 2012; Pisani et al., 2010). The resources at stake in this argument were access to good health services for populations and access to scientific resources for originating researchers in low- to middle-income countries (Sankoh & Ijsselmuiden, 2011; Tangcharoensathien et al., 2010). The main mechanism to support this principle was governance of data access decisions that adequately represent and balance stakeholders’ interests. Individuals’ choices to “opt out” of data sharing, as a component of a prior broad consent process, would also contribute to protecting their interests. Although some types of data were seen as less in need of careful governance (e.g., aggregated, fully anonymized, and “non-sensitive” data, such as demographic data), oversight was still necessary to define which data sets might be shared more openly. In any case, sensitivity was more often related to data use than data type.

The independence and make-up of a governance grouping were seen as critical to fairness of decision making. For community involvement, challenges in fair representation and enabling participation in often highly technical deliberations were recognized and remained unresolved. More consultation is likely to be needed to explore this area further. There was, however, clear agreement that communities of people whose data may be shared *should not be excluded* from processes of data sharing. Involving community stakeholders in developing responsive policies, as undertaken in this study, was an example of good practice seen as centrally important. A further potentially important suggestion was for regular feedback to community representatives and opinion leaders on data-sharing activities over time, to allow for community input to the types of decisions being made and amendment of data-sharing policies where needed. Given the importance of close familiarity with community interests, technical knowledge, and independence, experienced community liaison staff from other professional bodies in the country, such as non-governmental organizations, may also be able to “represent” the community in decision-making bodies.

A key recommendation was for the close involvement of national and local Ministry of Health partners in data-sharing governance. National-level Ministry of Health partners were seen as particularly important to provide checks and balances for international data sharing. Both local and national partners would be essential to actively promote near and long-term translational health benefits of research and clinical surveillance data sharing. There was some skepticism that government authorities would represent local interests. Yet governmental authorities are aware of existing general research governance policies, and the importance of a national framework for data sharing was repeatedly identified as important, particularly by community stakeholders.

A further good practice recommendation to support originating researchers, and indirectly primary communities’ interests, was sharing data within scientific collaborations between originating and requesting researchers (Pearce & Smith, 2011; Pisani et al., 2010; Sankoh & Ijsselmuiden, 2011). To be discussed in more detail in a future publication, this recommendation was strongly influenced by recognition of existing inequities in access to research and health resources for populations and researchers in low- to middle-income countries, where collaborative partnerships were seen as particularly important.

### Fair Processes and Trust

Taken together, the recommended forms of community involvement, informed consent, and governance of data sharing illustrate the role of trust in fair processes, given uncertainty that stakeholders’ interests will be represented in balancing benefits and challenges in future. It may be that the real risks of data sharing are often inflated (as some in this consultation suggested). However, a requirement for trust in research seems to be independent of risks in practice, particularly given the relatively novel nature of research data sharing to many researchers and all community stakeholders in this consultation.

Trust has been defined as a relational notion describing a voluntary act based on expectations of how other individuals or institutions will behave in future (Gilson, 2003). In this account, and strongly reflecting our findings, institutional forms of trust are likely to be strengthened by engagement and dialogue with citizens, and governance processes that include openness, solidarity, fairness, and truth-telling. Researchers in New Zealand have similarly pointed to the importance of independence, transparency, accountability, involvement of and feedback to community stakeholders or representatives, and working under national frameworks (Pearce & Smith, 2011). Other studies have highlighted the importance of building policies “bottom up” from public opinion in Canada (Hawkins & O’Doherty, 2010), of evenhandedness and accountability in sharing clinical trial data (Mello et al., 2013), and, for bio-repositories, of seeing governance as an opportunity to “align the interests of researchers with that of the community” (Fullerton et al., 2010, p. 2).

Key features of trust-building processes from this consultation included (a) making data sharing a “mutually acceptable enterprise”; (b) providing at least broad forms of prior informed consent, including rights to opt out of data sharing; (c) ensuring independence and accountability of governance processes; (d) providing feedback over time to communities; and (e) working within national frameworks. While policies to control future data use may risk being over-restrictive, our findings strongly suggest that these *managed* forms of access, developed through engagement with key local stakeholders, will be more likely to tip the balance in favor of ethical data-sharing practices (Pearce & Smith, 2011).

Over time, levels of concern about uncertainty among stakeholders in Kilifi may change, including where experience with data sharing increases. For different institutions with different types of relationships with study participants and “communities,” consultation outputs may also look different. For example, researchers based in referral hospitals with wide urban catchment areas may have different relationships with research “communities” to those interacting with focal and stable geographic communities. As highlighted throughout this special edition, more research in this and other settings is important to map public and “expert” opinion over time. An important component of such research would be building realistic understandings of benefits and risks of data sharing, including translational health benefits, such as direct impacts on policy and practice (Fullerton et al., 2010). Feeding this information back to researchers and community stakeholders may influence attitudes toward data sharing. Other centrally important areas for future research include developing effective and efficient methods for individual informed consent and community engagement, and community involvement in decision making and public awareness of data sharing that avoid tokenism. Our findings suggest that research in these areas is now a priority.

## Supplementary Material

Supplementary material

## Supplementary Material

Supplementary material
