# Supplementary material for: Involving Research Stakeholders in Developing Policy on Sharing Public Health Research Data in Kenya: Views on Fair Process for Informed Consent, Access Oversight, and Community Engagement
Source: J Empir Res Hum Res Ethics. 2015 Jul;10(3):264–77. doi: 10.1177/1556264615592385 (PMC4548475; doi:10.1177/1556264615592385)
Supplement: Supplementary material [file IDI_DS_v1.5_592385.pdf]

PHRDS Kilifi: Draft Interview guide

A. BACKGROUND

1. Which organisation are you currently working for? How long have you been working in this/these organisations?

2. What are your current professional role/responsibilities within this/these organisations, including specialist areas?

3. What types of research/specific projects have you been working on over the past 5 or so years?
   - main aims, scale, where conducted, types of participants, collaborators, funding
   - types of data collected
   - requirements of funders for data sharing?

4. What are you including here as 'data'? What *should* this include in your opinion/why?

B. GENERAL VIEWS ON DATA SHARING

5. From your general <u>reading/interactions</u> or any research you've been involved in <u>conducting</u> or <u>reviewing</u>, have you come across the concept of /been involved in 'research data sharing' between researchers? [*Probe for awareness of recent increased push from funders, journals and science standard setters to share research data more*]. For this work, 'data sharing' includes researchers sharing information they collect or generate during studies with other researchers. I will ask you more about any specific experiences later, but would first like to ask some general questions on your views about the idea of sharing public health research data.

6. What do you think about this 'policy' overall?
   - In general, what **advantages** (Box 6A) do you see for greater research data sharing? Who would this benefit and how? Would this directly benefit you at all?
   - **Any specific examples** in your experience? (?follow up section C)

   - In general, what **disadvantages** (Box 6D) do you see for greater research data sharing? Who would they affect, and how? Would these directly affect you?
   - **Any specific examples** in your experience? (?follow up section C)

7. Are there any types of data you think are most important to share, and why?
8. Are there any types of data we should be most careful about/avoid sharing altogether? What types, and what is the issue?

Do not prompt
For researchers, participants, community, others

6A: Scientific utility, integrity, cost effectiveness, other?
6D: confidentiality, benefit sharing, interests, autonomy, trust, other?

Throughout rest of interview, refer back to responses to these questions, and look for issues in Boxes 6A & 6D

C. SPECIFIC EXPERIENCES OF DATA SHARING

[*Where can talk about specific direct experience as primary researcher, requestor or reviewer; also from indirect experience/reading if no direct experience*] Can we now look in more detail at your experiences of/views on specific types of data sharing? Apart from those we've just talked about, do you have other experiences? *[Get overview of range and select 1 or 2 to discuss in detail, likely follow up on examples in Q6 but look for any use of KIDMS data].*

9. **General information** on data sharing experiences:
   - What was your role – reviewer, primary researcher, requestor, other?
   - What type of research and data were involved? Where were the data stored?
   - What was the main purpose of the request?
   - Who were the participants/data donors?
   - How did this request come about? E.g. was this a planned form of data sharing in the original protocol?
   - When was the data collected in relation to the request (e.g. less/more than 5 years?)

10. In this situation, what was the **process of data sharing**?
    - Who made decisions about whether the data could be shared and how [*national ERC/data governance committee, individual researcher, other*]? How?
    - **Were there any differences of opinion**? If so, what were these about, **how resolved** and what did you think about this process? Could it have been done in a **better way**?
    - Were there any forms/agreements/policy documents used? What were these? Are you able to provide copies?

***For Q 11-13 particularly look for references to issues in Boxes 6A & 6D*:**

Do not prompt
For researchers, participants, community, others

6A: Scientific utility, integrity, cost effectiveness, other?

6D: confidentiality, benefit sharing, interests, autonomy, trust, other?

11. We talked earlier about the benefits and potential problems involved in data sharing in gene these (or others) worked out **in this specific situation**? [*Probe again for issues in Box 6A/D*
    - What were the **advantages/benefits** of sharing this data, and to whom?
    - Were there any **issues/concerns** in sharing this data, what were these and who was primarily affected/how?
    - How did you feel about the **balance** between these benefits and issues in this situation? How comfortable/any sticking points for you?

12. Had the issue of **participants' consent** for data sharing been taken into account, and if so, how? [*Broad consent/tiered consent/not mentioned/other? If tiered, how?*] What did you think about this?

13. Given these issues, as a **primary researcher or requestor** (specify), did you feel you had any particular **responsibilities** to participants/researchers/requestor/others involved? What were these/to do what? How balanced where more than one set responsibilities/competing?
    - *[Probe for rights and interests of these parties, or wider scientific community/public]*
    - How strong did you think these responsibilities were e.g. an obligation or just to try to do your best?
    - Can you identify what particularly made you feel this responsibility?

14. What did you think about the data sharing process you experienced from a practical point of view – what was **straightforward or difficult**?
    - Challenges/facilitating factors?
    - What would you like to see changed, and in what way? Who should be involved in making these decisions? [*Probe for views on appropriate governance mechanisms for handling requests for data sharing of this type*]

D. KHDSS/CLINICAL DATABASE

Include where experiences did not include KIDMS data. Explain background to KIDMS where needed.

15. For the KHDSS/clinical database in Kilifi, what do you think about the importance of sharing this information with other scientists and any ethical challenges this might involve? [*Using examples of types of data sharing requests in the past and in future, probe for perceptions and experiences of issues for & against sharing data in this situation, and reasons for these views. Refer Boxes A & D]*

16. What do you think is the best way for decisions to be taken about requests from other researchers for access to data in this database? Who should be involved in making these decisions? Why? [*Probe for views on appropriate governance mechanisms for handling requests for data sharing]*

E. GENERAL CONSENT AND GOVERNANCE PROCESSES

17. **Data sharing governance**: Are there any processes in place in your organization to make decisions about what and how data collected by internal researchers should be shared: with other internal researchers and with external researchers? What are these? [*e.g. In Kilifi, DGC/DS policy*]
    - What was the process of setting this up – who was involved? Should others have been involved? Who?
    - Who is involved in making decisions about data sharing? Who do you think should be involved, and why? *[Probe for participants/community representation/researchers/funders/independent experts/others]*
    - Are there situations where you think that explicit governance of data sharing may not be necessary (anonymised data, long-archived data, between close collaborators)
    - What do you think are the advantages and disadvantages of these data governance policy/processes? How improved? What would you prefer to see in place (including none)? Why? (if not clear)

18. **Influence of context on researchers' responsibilities**: In general, how might the **details** of a request influence your view on researchers' responsibilities around data sharing *[to science/science institutions/funders/other researchers/participants]*? ie. Do you feel that differences in the following could change these responsibilities, and how? - the type of data/research; the participants involved; the requestors; the purpose of the request; the type of consent given; the time since data collected? Something else? In what way?

19. We have talked about some of the challenges in asking for consent for future use of data and you described the way the consent process worked in a particular example (Q12). Ways that have been recommended for consent processes in this situation are: **broad consent; tiered consent; and re-consenting** for all future use. [*Explain as needed*]
    - What do you think about these types of processes overall - the advantages and disadvantaged?
    - In what situations should they be used?
    - For tiered consent, what kinds of constraints do you think participants might want to put on the use of data? *[Geographical/types of research or disease/who can access?]*
    - Are there situations where explicit consent for data sharing may not be necessary (anon/long standing data)
    - What process would you prefer to see in place in your organization (including none)? Why? (if not clear)

20. What do you think about using the **term 'data sharing' in a Kenyan context** to describe the movement of data between researchers? How does this translate in Swahili & culturally? Does the term 'sharing' have connotations about relationships that make it appropriate or would there be a better way of conceptualizing this policy?

21. What kind of **outputs** from this project might be a **useful resource** for you in your work?

F. SHARING DATA FROM THIS INTERVIEW

22. [*Remind about consent process at start of this interview, including plans for data sharing*]. Now that you've completed the interview, how do you feel about the information you've given me (once anonymised) being made available to other researchers who are working on the same topic, but maybe in different countries, now and in the future?
    - How would you feel about this if the information was given: as summary reports across all interviews; as summary reports including anonymised quotations; as sections of transcripts; as whole transcripts?
    - For summary reports, would you prefer to see quotations before these are shared?

For each participant, document following information:

Interviewer initials/Date interview/ Code/ Name/Organisation/Professional role/ Age/ Sex/Religion/Nationality/Place/ Time start & end/Comments on interview
