# Supplementary material for: Involving Research Stakeholders in Developing Policy on Sharing Public Health Research Data in Kenya: Views on Fair Process for Informed Consent, Access Oversight, and Community Engagement
Source: J Empir Res Hum Res Ethics. 2015 Jul;10(3):264–77. doi: 10.1177/1556264615592385 (PMC4548475; doi:10.1177/1556264615592385)
Supplement: Supplementary material [file PHRDS_FGD_guide_v4_3_582385.pdf]

PHRDS Kilifi: FGD topic guide

A. Introductory session - 25min

Introductions, explain study, consent & start voice recorder. THIS STUDY IS ABOUT WHEN & HOW DATA FROM STUDIES CAN BE SHARED WITH OTHER RESEARCHERS – WHAT DO DIFFERENT PEOPLE THINK?

General discussion on 'what is KEMRI' (partnership with MoH) and 'what is research'. How proposals are developed and reviewed. Explore experiences/perceptions of different types of research done at KEMRI Kilifi – establish that many different types are done.

Explore perceptions of <u>what kinds of information</u> researchers collect during studies. Introduce idea that many different types of information are collected or generated during research [note this is about data not samples]:

- People are asked questions – the information is recorded individually
- Blood samples are taken for tests – the test results are recorded as individual information
- Individual information is looked at across all participants as part of research. This grouped (aggregated) information is about populations, not individuals. e.g. rates of bed net use
- When information is collected during studies, researchers often also do more work that 'generates' new types of data e.g. when researchers do special tests on blood samples to find out how people fight malaria as they get older (looking at the substances that are produced when the body fights in this way) - some research information is '<u>collected</u>' and some is '<u>generated</u>' by researchers using the information they collect.

* Data used for primary research and secondary research also.
*Research data different from research findings.

B. Sharing background information/exploring views on census & KCH surveillance data 40min

1. **Explore knowledge & attitude to KHDSS DATA using open questions and sharing information to build understanding in box below, using visuals:** One type of research that KEMRI carries out is through visiting all the households in this area to collect different types of information about the people who live there ('census').

   What do you know about this work? *Probe for*:
   - What information collected? [*Probe for all types KHDSS info known about*]
   - What is purpose of collecting information? [*Probe for any use data, including public health & research; and for any ideas about how different types information used*]
   - Who uses it in this way? Which organisations involved? [*for use by KEMRI/MoH/other*]

   What are your views about this work? *Probe for:*
   - General views – positive and negative – based on perceptions or experiences
   - What types of information collected in census and KCH surveillance are sensitive, and why?

<u>About the census</u>:

- **Purpose:** Set up to support <u>KEMRI to conduct research</u> and <u>MoH in Kilifi to monitor health and plan PH programmes</u> for this and similar communities. Give concrete examples of PH and research:
  - Supporting MoH: e.g. catch up polio vaccination programme from information on immunisation status
  - Supporting research: e.g. random sampling for a study – make sure findings apply to all people in future
- **How it works:**
  - Types data collected: every 4 months, residents, births, deaths, pregnancy information, age, gender, household structures, GPS, plus extra information from time to time (water sources, immunisation)
  - *Link to 'types' of data in introduction*
  - One of many MoH & health research censuses in Africa & rest of world, set up for similar reasons
  - Verbal consent from HHH on behalf of rest of family

2. Share information on KCH clinical surveillance data collection and use; & link to KDHSS data to build understanding in box below, using visuals and asking open questions where possible:

1. <u>About linkage between census and hospital surveillance data if admitted</u> *(draw on group if possible)*

- In Kilifi, <u>information from census can be linked to routine information collected on patients on wards</u> at KCH ('patient information') **at time of any future hospital admission.**
- <u>Routine patient information</u> includes symptoms, examination findings, results of tests such as x-rays or blood tests (including HIV where done), diagnosis, treatment, response to treatment & how long on ward.
- *Link to 'types' of data in introduction*
- Here in Kilifi therefore we a have a large data set of this linked information that we have accumulated for the last 10 years and we continue to collect such new information.
- Purpose of link: Done to support KEMRI research and MoH monitoring and planning for services in Kilifi, including many years in the future:
  - Supporting MoH: e.g. if there is a cholera outbreak, MoH staff can quickly identify where in Kilifi hospital cases have come from to begin control measures in community (like treating wells)
  - Supporting research: e.g. in testing new malaria medicines, researchers can easily follow children up at home after discharge from hospital
  - May be done many years later (even more than 10 years later!).
  - It is important to know that data that is stored in this way and that from research may be used to further conduct other research and new questions asked using the data that was generated from the initial research (we refer to this as secondary research).
  - Information on password protected computers in ICT in Kilifi
  - <u>ALWAYS</u> **names (& other identifiers e.g. addresses) removed and replaced with codes (could be re-linked for special purposes and with special permission)**

2. <u>About wider potential use of anonymised linked KHDSS and KCH information in research</u>

- In the same way that researchers in Kilifi use KHDSS/KCH information as part of many different studies, <u>other researchers outside Kilifi</u> would also find it useful to do research using the data from Kilifi
- **In fact, everywhere in the world, researchers and organisation that fund research have realised that 'sharing' the information collected or generated during research activities can help others to do more research, and generate more new knowledge, more efficiently.**
- This is only done AFTER any individual identifying information removed (e.g. names, addresses etc)
- Some reasons sharing research data seen as positive are:
  - Being more efficient than conducting new studies when the information already exists
  - Letting researchers from different places bring together information to ask different questions
  - Making sure participants' contributions are used as efficiently as possible – causing less inconvenience to future participants?
  - Letting researchers 'check' each other's results
- Could be at any time – within a year, after 10 years or even more

In remainder of discussion, talking about your views on this – including looking at 'pros and cons'

TEA BREAK

C. Introducing & discussing scenario of data sharing: Progressive probing – 1hr

We would like to find out more about your views on when and how researchers should agree to share information they collect or generate during studies with other researchers. **To do this, we are going to give you an imaginary scenario to think about first. We will then later change different aspects of the scenario and see if you think this makes any difference to your views.**

Explain basic scenario: A researcher working with another government research institution in Kenya needs data to try to find out if there is any association between having good access to water supplies and chances of being admitted to hospital for diarrhoea. Results from this work will help to come up with better ways of preventing diarrhoeal diseases in the community the researcher is working with. KWTRP has been collecting this information for a long time in Kilifi, where researchers use it to conduct different types of research while KCH uses it to carry out several interventions (as Salim said). The researcher is working in an area which is very like Kilifi but do not have a census or hospital surveillance system. The information they want is about all individuals admitted to KCH with diarrhoea in a one year period (Remember: after taking any identifiers - like names - off & replacing with codes):

- all the clinical information about their admission
- what kind of water supply they have at home (from census)

* Data to be shared, not research findings.

(Check if any clarification needed – ask open questions to see if have understood scenario)

3. **Explore views: Do you think researchers should agree** to give the information researchers have asked for? Why or why not? [*Seek views from all participants, focus on purpose, encourage discussion*]
    - What reasons to share/not share information in this way? [*Look for issues in Box A & D*]
    - Where think should not share, is there anything that researchers could do to make this possible or should they just say no?
    - *If consent/awareness seen as important/condition, ask*: Why important? If information is completely anonymised (name/place/role taken off) and purpose cannot harm individuals/community who gave information in any way, do you still think awareness always important? [*Probe for right to know*]

IF CONSENT DISCUSSED HERE, INTRODUCE Q9…….>

4. **Is there anything about the researcher or where they are working or anything else that would make you feel differently about this application? What and why?** [*Seek views from all participants, encourage discussion]*
    - Probe for any factors that would change people's views, including - **type** of institutions (another research centre or a University in Kenya? An NGO or CBO in Kilifi?) AND **where based** (Kilifi/ Coast/elsewhere in Kenya/outside Kenya/East/West/Other parts Africa & USA/Europe/China?) OR **anything else** [*Probe for issues of trust, collaboration, conflicts of interest*]

5. **Does time matter?** If request made one year or 10 years after data collected, does this make a difference? What & why? Is there another duration that is important e.g. much longer?

6. **What if the researchers were seeking information for a research project on a topic NOT LIKELY to benefit many people living in Kilifi or similar settings?**
    - Would this make a difference to your view? How/why? What reasons for sharing/not sharing? [*Probe for issues of benefits for 1st community; trust/collaboration; time frames; global vs local]*
    - *If needed, give this e.g. We know that when people are very overweight they can have health problems such as high blood pressure, heart disease, joint disease and some types of cancer. But not many people in Kilifi are very overweight – being underweight is more a health problem here. So what if the researchers wanted to do a study on health problems linked to being very overweight?*

❖ *If participants change their minds from previous viewpoint on data sharing, explore without criticising.*

Summarise points made so far about reason for/concerns about sharing information, & add as needed:

Main reasons for:

- Learning more from information collected/generated
- Possible for researchers' work to be 'checked' by others?

Main concerns that:

- People who gave the information might be identified or 'stigmatised' in any way, depending on data shared?
- People should understand how the information they gave will be used, even if anonymised?

D. Talking about consent & governance – 45min

7. **Follow up On previous section to explore views about how to ask for consent for future use when this is not possible to specify***:* Not easy to ask participants for permission to share information in future since don't necessarily know what these future requests might be...does this matter and what should be done? *Probe for following types of ideas:*
    - It's enough to make sure people know when they give the information that it may be used in future for different studies, without detailed information about those studies
    - It's enough to make sure people give permission for future use (as above) but also should be some way of checking/safeguarding the interests of people/communities who gave information at the time the new request is made (refer 'concerns' in Q18 and follow up in Q20). ***Link to Q12 if raised.***
    - It's important to go back to individuals in future to ask specifically about any new uses in future i.e. revisit homes etc. (practical challenges?)
    - Other? E.g. community engagement as support

8. **About anonymisation**: In above discussion, what do you see as the importance of 'anonymising' (taking people's names off)? *[Probe for how this affects views on importance of consent and ideas about 'ownership']*

9. **If a safeguard needed**, some people have said this should be some type of committee to look at future requests. If you think this would be useful, which types of people do you think should be included in this committee – in order to make you feel confident about how information would be used in future? *[Probe for technical experts, independent persons and community representatives – which type?]*

10. **Sensitive information**: Which kinds of information collected in research (including clinical surveillance, KHDSS and other studies people know about) do you think are particularly sensitive such that people will think twice before they give such information or they will be uncomfortable to share?, And in what way? How does this affect your views on data sharing/consent/governance? *[Listen to all views then ask specifically about: identifying, ethnicity/ religion, HIV, sexual behaviour]*

………..

Issues to look for in all discussions (avoid direct prompting as far as possible)

A: Reasons to share

For participants:

- Better use made of their contributions to research?

For researchers:

- Answer more/bigger questions with same data
- Saves collecting data again
- Less resources needed for research

Others – for anyone?

D: Reasons not to share

For participants/community:

- Risks of individual/group identification and therefore harm
- Risks to individual/group identification anyway wrong
- Individual/community should benefit
- Individual/community has right to know data will be shared (so only with some form of consent)
- Individual's contribution should be recognised
- Risks to trust

For researchers: Recognition/career development/(IPR)

Others – for anyone?
